# The complete mitochondrial genome of *Engraulis ringens* (Engraulidae, Clupeiformes) and phylogenetic studies of Engraulidae

**DOI:** 10.1080/23802359.2019.1675553

**Published:** 2019-10-11

**Authors:** Wei Sun

**Affiliations:** Department of Automobile Engineering, Weifang Engineering Vocational College, Qingzhou, P. R. China

**Keywords:** *Engraulis ringens*, mitogenome, phylogenetic tree

## Abstract

In this study, the complete mitochondrial genome of *Engraulis ringens* has been determined. The whole sequence is 16,690 bp in length and included the typical structure of 13 protein-coding genes, 22 tRNA genes, 2 rRNA genes, and 2 non-coding regions (control region and L-strand replication origin). The overall base composition includes A 26.65%, T 26.09%, C 28.13%, G 19.13%, with a slightly A + T bias of 52.74%. Moreover, the 13 PCGs encode 3797 amino acids in total. The phylogenetic analysis of *E. ringens* in the Engraulidae family was constructed based on 12 PCGs encoded by the heavy strand, and the result showed that *E. ringens* is most closely related to *Engraulis japonicus* and *Engraulis encrasicolus*.

The Peruvian anchoveta (*Engraulis ringens*) belongs to Engraulidae, Clupeiformes. They are mainly found in the Southeast Pacific Ocean near Peru and Chile. They have a large catch and may be the most abundant fish in the world, mainly for fish meal industry (Cury et al. 2000). Due to overfishing and El Niño, the number of anchovies has been greatly reduced (Barber and Chavez 1983; Bertrand et al. 2004). Considering its economic value, it is necessary to obtain its molecular information. We described the complete mitogenome of *E. ringens*, studied the phylogenetic relationship within Engraulidae, and provided new ideas for the breeding of this species.

*Engraulis ringens* was collected from the South China Sea (23°34′95ʺN; 116°78′57ʺE) and stored in the laboratory of Weifang Engineering Vocational College with the accession number 20180405BL13. Total genomic DNA was extracted from the muscle tissues of individual specimens by using the phenol-chloroform method (Barnett and Larson 2012). The complete mitochondrial DNA sequence was first determined using PCR and sequencing. Sequence alignment was conducted by CodonCode Aligner. MEGA 6.0 software was used to calculate base composition and build the phylogenetic tree (Tamura et al. 2013).

The complete mitochondrial genome of *E. ringens* is 16,690 bp in length (GenBank accession No. MH732975), consisting of 13 protein-coding genes, 22 transfer RNA genes (tRNA), 2 ribosomal RNA genes (12S rRNA and 16S rRNA), one L-strand replication origin (OL), and one control region (D-loop) (Boore 1999). The overall base composition is A 26.65%, T 26.09%, C 28.13%, G 19.13%, respectively. The A + T content (52.74%) is higher than G + C content, which is similar to other Engraulidae mitogenomes (Bo et al. 2013; Li et al. 2016; Wang et al. 2016; Zhang et al. 2016; Jiang et al. 2018). ND6 and eight tRNAs (Gln, Ala, Asn, Cys, Tyr, Ser, Glu, Pro) were encoded by the L-strand, other genes were encoded by the H-strand. Twelve protein-coding genes start with ATG except COI (GTG) (Miya et al. 2001). Eight PCGs (ND1, COI, ATP8, ATP6, COIII, ND3, ND4L, and ND6) typically terminate with TAA as the stop codon; two PCGs (ND2 and ND4) end with TA–; three PCGs (COII, ND4, and CYTB) ends with T––. The 12S rRNA (952 bp) is located between tRNA^Phe^ and tRNA^Val^ genes, and 16S rRNA (1,699 bp) is located between tRNA^Val^ and tRNA^Leu^ genes. The control region (D-Loop) is located between tRNA^Pro^ and tRNA^Phe^ genes and is 1029 bp in length.

Phylogenetic tree (Figure 1) was constructed using the neighbour joining (NJ) method based on the 12 protein-coding genes encoded by the heavy strand of 16 Engraulidae species. The result showed that *E. ringens* first clustered with *Engraulis japonicus* and *Engraulis encrasicolus*, suggesting a very close relationship of these three species.

**Figure 1.**
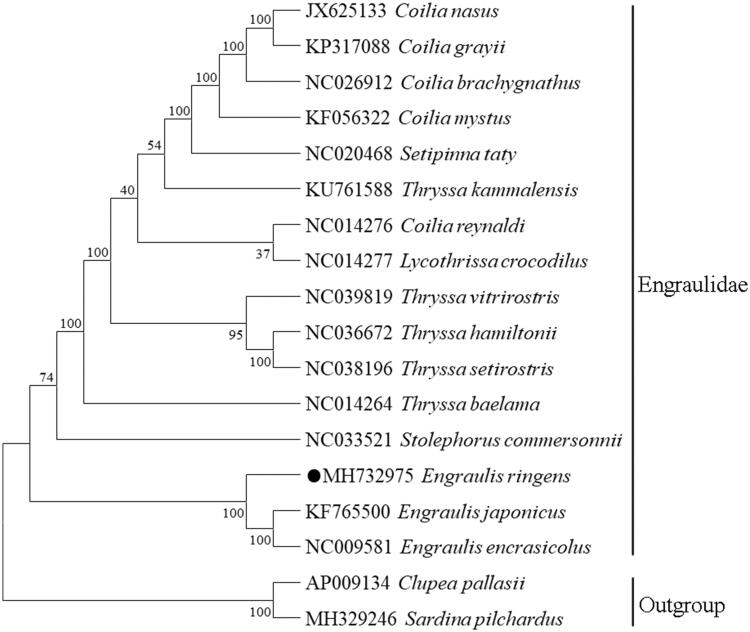
Neighbour Joining (NJ) tree of *E. ringens* and other 15 Engraulidae species based on 12 PCGs encoded by the heavy strand. The bootstrap values are based on 1000 resamplings. The number at each node is the bootstrap probability. The number before the species name is the GenBank accession number. The genome sequence in this study is labelled with a black spot.
